# Discovery of the *REN11* Locus From *Vitis aestivalis* for Stable Resistance to Grapevine Powdery Mildew in a Family Segregating for Several Unstable and Tissue-Specific Quantitative Resistance Loci

**DOI:** 10.3389/fpls.2021.733899

**Published:** 2021-09-03

**Authors:** Avinash Karn, Cheng Zou, Siraprapa Brooks, Jonathan Fresnedo-Ramírez, Franka Gabler, Qi Sun, David Ramming, Rachel Naegele, Craig Ledbetter, Lance Cadle-Davidson

**Affiliations:** ^1^School of Integrative Plant Science, Cornell AgriTech, Cornell University, Geneva, NY, United States; ^2^BRC Bioinformatics Facility, Institute of Biotechnology, Cornell University, Ithaca, NY, United States; ^3^United States Department of Agriculture (USDA)-Agricultural Research Service (ARS), Grape Genetics Research Unit, Geneva, NY, United States; ^4^United States Department of Agriculture (USDA)-Agricultural Research Service (ARS), Commodity Protection and Quality Research, San Joaquin Valley Agricultural Sciences Center, Parlier, CA, United States; ^5^United States Department of Agriculture (USDA)-Agricultural Research Service (ARS), Crop Diseases, Pests and Genetics Research Unit, San Joaquin Valley Agricultural Sciences Center, Parlier, CA, United States

**Keywords:** grapevine powdery mildew, *Erysiphe necator*, amplicon sequencing, quantitative resistance, race specific resistance

## Abstract

Race-specific resistance loci, whether having qualitative or quantitative effects, present plant-breeding challenges for phenotypic selection and deciding which loci to select or stack with other resistance loci for improved durability. Previously, resistance to grapevine powdery mildew (GPM, caused by *Erysiphe necator*) was predicted to be conferred by at least three race-specific loci in the mapping family B37-28 × C56-11 segregating for GPM resistance from *Vitis aestivalis*. In this study, 9 years of vineyard GPM disease severity ratings plus a greenhouse and laboratory assays were genetically mapped, using a rhAmpSeq core genome marker platform with 2,000 local haplotype markers. A new qualitative resistance locus, named *REN11*, on the chromosome (Chr) 15 was found to be effective in nearly all (11 of 12) vineyard environments on leaves, rachis, berries, and most of the time (7 of 12) stems. *REN11* was independently validated in a pseudo-testcross with the grandparent source of resistance, “Tamiami.” Five other loci significantly predicted GPM severity on leaves in only one or two environments, which could indicate race-specific resistance or their roles in different timepoints in epidemic progress. Loci on Chr 8 and 9 reproducibly predicted disease severity on stems but not on other tissues and had additive effects with *REN11* on the stems. The rhAmpSeq local haplotype sequences published in this study for *REN11* and Chr 8 and 9 stem quantitative trait locus (QTL) can be used directly for marker-assisted selection or converted to SNP assays. In screening for *REN11* in a diversity panel of 20,651 vines representing the diversity of *Vitis*, this rhAmpSeq haplotype had a false positive rate of 0.034% or less. The effects of the other foliar resistance loci detected in this study seem too unstable for genetic improvement regardless of quantitative effect size, whether due to race specificity or other environmental variables.

## Introduction

Traditionally, quantitative resistance (QR) has been of interest to plant breeders to provide more durable resistance than qualitative gene-for-gene resistances that are often race-specific (Poland et al., 2009). However, while some QR loci confer broad-spectrum resistance, others confer race-specific resistance. For example, when challenged with three isolates of *Pyricularia grisea*, nearly all QR loci were race-specific, effective against only one or two isolates: 10 of 12 QR loci in a rice population and 11 of 12 QR loci in a barley population (Chen et al., 2003).

At least 13 resistance loci have been characterized against GPM (caused by *Erysiphe necator*), (http://www.vivc.de/index.php?r=dbsearch%2Fdataonbreeding). Few confer qualitative resistance that almost completely restricts pathogen colonization [*RUN1, REN4, REN6*, and possibly *REN5* (Blanc et al., 2012)], and the remainder confer QR of minor-to-moderate effect (Cadle-Davidson, 2019). Regardless of effect size, with the exception of *REN4*, all GPM resistance loci characterized in-depth for race specificity have presented at least preliminary indications of being race-specific (Cadle-Davidson, 2019), even resistance sources for which the genetic basis has not to be mapped (Ramming et al., 2012; Barba et al., 2015). Diverse isolates of *E. necator* have been used to inform strategies for which race-specific genes are complementary and, thereby, may enhance durability (Feechan et al., 2015). In grape breeding programs, these resistance loci are being tracked and stacked primarily with microsatellite markers (Di Gaspero et al., 2012; Pap et al., 2016; Zendler et al., 2017), single nucleotide polymorphism (SNP) markers (Myles et al., 2015; Yang et al., 2016), and/or an integrative amplicon sequencing (AmpSeq) marker multiplex (Fresnedo-Ramírez et al., 2017).

Previously, resistance to GPM derived from the interspecific *Vitis* hybrid cultivar “Tamiami” was shown to be race-specific and was suggested to be governed by at least three genetic loci, based on F_1_ family segregation ratios (Ramming et al., 2012). In a vineyard in Parlier, CA, Tamiami pseudo-testcross progeny segregated 7:1 or 15:1, indicating at least three functional resistance genes, any of which were sufficient for vineyard resistance. In contrast, the same progeny in a nearby greenhouse or controlled inoculation segregated 1:1, indicating one functional resistance gene. This suggested that most of the Tamiami-derived resistance loci were race-specific and only effective in the vineyard. While the conclusions supported qualitative resistance loci, this could be an artifact of applying thresholds to convert quantitative traits to qualitative traits for Mendelian analysis. The cellular resistance phenotype was characterized by a mild reduction in successful penetration and colony formation, visible epidermal cell necrosis subtending appressoria, and a strong reduction in secondary hyphal length. Controlled inoculation using isolates adapted on *V. aestivalis* or *V. aestivalis*-derived “Norton” were able to infect resistant seedlings and sporulate, confirming race specificity (Ramming et al., 2012).

Recently, a set of 2,000 DNA markers targeting the *Vitis* core genome were designed and implemented using rhAmpSeq chemistry (Zou et al., 2020). These markers were shown to be highly transferable across the diversity of *Euvitis*, meaning that most of the markers return data regardless of the genetic background, so results can be shared between research programs and breeders. This core genome marker set amplifies 250 bp local haplotypes that span multiple SNPs and/or Indels, resulting in enriched information content to track ancestral haplotypes and recombination (Zou et al., 2020). This rhAmpSeq core genome marker set is being used for genetic mapping and marker-assisted selection across grapevine breeding programs.

In this study, the previous hypothesis that “Tamiami” confers three or more independently assorting loci for GPM resistance (Ramming et al., 2012) was tested, using a multiyear vineyard evaluation to track the reproducibility of resistance results across environments and on four tissue types. In addition to a categorical 4-point severity scale, we used a quantitative severity measure [area under disease progress curve (AUDPC)] to gain insights into the quantitative effects of these resistance loci. We used the rhAmpSeq core genome marker strategy to generate a genetic linkage map and analyzed extensive phenotype data to identify markers linked to resistance from “Tamiami” in two related families.

## Methods

### Germplasm

*Vitis* hybrid cv. Tamiami is an F_1_ hybrid resulting from the cross *V. aestivalis* “Fennell 6” × *Vitis vinifera* “Malaga.” In 2002, the segregating modified-BC_1_ (mBC_1_) 02-3512 family with “Tamiami” resistance was developed by crossing B37-28 (“Tamiami” × *V. vinifera*) × C56-11 (*V. vinifera*), resulting in 94 full-sibling progenies. The cross was repeated in 2007, resulting in “Tamiami” derived family 07-3509 with 150 full-sibling progenies. To validate quantitative trait locus (QTL) results in an independent family, a third cross was made “Tamiami” × *V. vinifera* “Solbrio,” resulting in 300 full-sibling progenies. C56-11 and Solbrio are susceptible *V. vinifera* breeding parents in the USDA- Agricultural Research Service (ARS) grape breeding program at Parlier, CA.

### Parlier, CA Vineyard Phenotyping

As previously described, the progeny of 02-3512 were grown at USDA, ARS San Joaquin Valley Agricultural Sciences Center, Parlier, CA, on their roots at 4 m × 0.5 m spacing and were cane pruned on a single T-trellis (Ramming et al., 2012). No fungicides were applied. Highly susceptible *V. vinifera* cv. Ruby Seedless plants were planted in every 15th vine as an inoculum source and to check for the amount of natural GPM infection. Once the plants started fruiting (in their 3rd growing season), natural GPM severity assessments were performed between July and October for 9 years, often with two ratings per year: an “early” rating after susceptible “Ruby Seedless” vines had >50% foliar coverage and a “late” rating 2 or 2 months later. Foliar disease severity ratings were recorded based on percent of foliage covered by GPM colonies: 1 = no visible infection; 2 = very few small colonies; 3 = <50% coverage; 4 = >50% coverage. Analogous ratings were recorded for three other tissues: rachis, berries, and stems.

To evaluate resistance more quantitatively AUDPC values were calculated for the same planting in 2016 and 2017 based on bi-weekly ratings using a modified Horsfall-Barratt scale (Horsfall and Barratt, 1945). Specifically, leaves, rachis, berries, and stems each were evaluated on a 1 to 9 scale: 1 = no disease, 2 = 0 <2.5%, 3= 2.5 <10%, 4 = 10 <20%, 5 = 20 <25%, 6 = 26 <45%, 7 = 45 <60%, 8 = 61 <80%, 9 = 81 <100%. For each vine, AUDPC was calculated by taking the average disease rating of two sequential time points multiplied by the time interval between those points and calculating the total sum across all time intervals (Shaner and Finney, 1977).

### Parlier Greenhouse Phenotyping

Progeny of 02-3512 was screened for resistance to GPM as young vines grew from dormant cuttings in USDA-ARS, Parlier, CA greenhouses as described previously by Ramming et al. (2012). Vines were grown in 6.35 cm^2^ × 17.8 cm high Anderson pots (Anderson Die and Manufacturing, Portland, OR). Two GPM susceptible *V. vinifera* “Ruby Seedless” vines were placed in the middle of each tray to provide the natural inoculum source surrounded by 13 randomly placed test vines. Symptoms were evaluated when 70% of susceptible control “Ruby Seedless” leaves exhibited conidiating colonies. After the first evaluation in September 2006, the epidemic was allowed to progress further for the second evaluation in November 2006. The presence of mycelia was confirmed by microscopy. Incidence (percentage of leaves with one or more GPM colonies) and severity (percentage of foliage covered by GPM colonies) scores were recorded separately, and then were averaged.

### Laboratory Phenotyping Via Hyphal Transects

To phenotype resistance in controlled experiments as described previously in detail by Cadle-Davidson et al. (2016), one leaf from each of four replicate shoots was sampled at the 3rd node and represented as two replicate surface-sterilized disks on eight agar trays, each a replicate block containing all progeny and check samples. *E. necator* isolate NY90 (originally from *V. vinifera* “Chardonnay” in Burdett, NY; Barba et al., 2015) was inoculated at 2 × 10^5^ conidia/ml in 0.001% Tween-20 using a handheld atomizer (Preval, Coal City, IL). Inoculated samples were maintained at 23°C in a 12-h photoperiod for 9 days until processing. Samples were cleared in 3:1 v/v ethanol:acetic acid until the tissue was completely bleached, then stained with Coomassie Brilliant Blue R-250 (Bio-Rad Laboratories, Richmond, CA), and mounted for hyphal transect data collection using a compound microscope. Hyphal transects count the number of interceptions of individual hyphae crossing one of two axial transects (vertical and horizontal) of the entire disk at X 200.

### Genotyping

DNA of 02-3512 and 07-3509 families was extracted from 1 cm or smaller leaves using QIAGEN DNeasy 96 Plant Kits with 3% w/v PVP40 added to the lysis buffer. DNA of “Tamiami” × “Solbrio” and the rhAmpSeq diversity panel were extracted from 1-cm or smaller leaves by Intertek AgriTech (Intertek ScanBi Diagnostics, Elevenborgsvägen 2, Alnarp, Sweden) using an automated magnetic bead pipeline with sbeadex kit and a PVP-amended buffer provided by LGC (Teddington, United Kingdom). DNA was used without within-plate normalization but was quantified by QuantiFluor fluorescence as per the instructions of the manufacturer (Promega, Madison, WI) and diluted to target about 30 to 60 ng of template DNA, based on the average concentration of eight quantified samples. rhAmpSeq amplification enrichment using the 2000 marker panel was conducted following the protocol of the manufacturer (IDT-DNA, Redwood City, CA), but with half-volume reactions (Zou et al., 2020). In brief, the first polymerase chain reaction (PCR) used 14 cycles with annealing temperature at 61°C for each sample. The PCR products were diluted at 1:20 and indexed with Integrated DNA Technologies, Inc (IDT), Redwood City, CA, USA indexing primers using 24 cycles with an annealing temperature at 60°C. The indexed PCR products were pooled, cleaned with Agencourt AMPure beads, quantified, and sequenced on an Illumina (Illumina, San Diego, CA, USA) NextSeq (2 × 150 bp) sequencer.

rhAmpSeq sequencing data were analyzed using the *analyze_amplicon.pl* Perl script (https://github.com/avinashkarn/analyze_amplicon/blob/master/analyze_amplicon.pl; Zou et al., 2020) to obtain haplotype variants (per marker) across all vines in each family, to generate the haplotype to genotype (*hapgeno*) file. Monomorphic markers and markers with >75% missing data in the *hapgeno* file were manually removed from further analyses. Finally, using a custom Perl script, *haplotype_to_VCF.pl* (https://github.com/avinashkarn/analyze_amplicon/blob/master/haplotype_to_VCF.pl; Zou et al., 2020), the four most frequent haplotype alleles for each marker (representing the four possible haplotypes within a heterozyogous diploid bi-parental family) in the *hapgeno* file were converted to a variant call format (VCF) file, where each haplotype allele of a marker was converted to a pseudo A, C, G, or T allele, for further marker validation analyses that are discussed hereafter. Genotype data are available in NCBI BioProject PRJNA281110.

### Genetic Map Construction

For each grapevine family, the VCF file was imported into Trait Analysis by aSSociation, Evolution and Linkage (TASSEL) version 5.2.51 software (Bradbury et al., 2007) for genotype imputation using the default parameters of the LD-kNNi imputation plugin, also known as LinkImpute (v1.1.4) (Money et al., 2015) as previously described by Zou et al. (2020). Post-imputation, vines with >90% missing data were removed from the analysis. Quality control focused on the removal of vines with genotypes appearing to be due to self-pollination, pollen contamination, or other contaminants. Post-imputed and filtered markers were used to calculate a genome-wide pairwise identity-by-state (IBS) distance matrix for each family in TASSEL software using 1–IBS, followed by multidimensional scaling (MDS) analysis (Bradbury et al., 2007), and the first two principal coordinates were graphically depicted using the R statistical software. Vines not grouping with full-sibling progeny were removed from linkage mapping analysis (Supplementary Table 1). In addition, based on parental alleles, for each progeny vine, we calculated the percentage of loci inconsistent with Mendelian expectations using bcftools plugin *mendelian* (cite), and the outlier vines with high-mendelian inconsistencies (Supplementary Table 2) removed from the downstream analyses.

Genetic maps were constructed in Lep-MAP3 v.0.2 (LM3; Rastas, 2017) as previously described by Zou et al. (2020) using the VCF file of the post-imputed and filtered markers and curated full-siblings, with the following LM3 modules: (1) *ParentCall2* to call parental genotypes; (2) the resulting output was filtered by using *Filtering2* (parameter *dataTolerance* = 1.00E-6) with markers filtered out based on monomorphism or a two-sided χ^2^ (chi-squared) test (testing if the allele ratio has significantly deviated from the expected mendelian ratio, at the above tolerance thresholds); (3) *SeparateChromosomes2* module was used to identify linkage groups; and (4) *OrderMarkers2* to compute the sex-specific parental genetic distances of the markers in each linkage group using 20 iterations per group. Correlations of genetic and physical distances of individual markers per chromosome were plotted to evaluate the consistency of the maps, genome organization, and structural variation.

### QTL Analysis

The phased output data from *OrderMarkers2* were converted into genotypes (1 1, 1 2, 2 1, 2 2) using *map2genotypes.awk* script and further into R/qtl 4-way cross-format, where,1 1 = AC = 1, 1 2 = AD = 2, 2 1 = BC = 3 and BD = 2 2 = 4. QTL mapping was performed in the statistical software R version 3.6.3 (2020-02-29) using the *qtl* package (Broman et al., 2003) focused on the sex-averaged genetic map, assuming a common recombination rate in both the parents. Genotype probabilities were calculated using *calc.genoprob* with step = 0 (probabilities were calculated only at the marker locations) and assumed genotyping error rate of 1.0E-4. The *scanone* function was implemented to perform standard interval mapping with Haley-Knott regression (Haley and Knott, 1992). Logarithm of the odds (LOD) significance thresholds were determined by 1,000 permutation tests at an alpha of 0.1 to detect minor QTLs. QTL support interval was determined calculating 1.5—LOD support intervals by using *lodint*. The percentage of variance explained in the context of a full additive model was calculated using *fitqtl*.

### Statistics

Pearson Correlation was calculated for AUDPC severity ratings among tissues in 2016 and 2017 using the *PerformanceAnalytics* package, and distributions were plotted using the *ggplot2* package in R software version 3.6.3 (2020-02-09). Best linear unbiased estimates (BLUEs), also known as least squares, were calculated to avoid shrinkage toward the mean using the *glm* function for the vines for each trait in Trait Analysis by aSSociation, Evolution and Linkage (TASSEL) version 5.2.51 software, where years were included in the model as main effects and the effects of vines as fixed.

The presence/absence of the resistance locus *REN11* among the germplasm was determined by the Bayesian hypothesis testing, with a null hypothesis that no resistant allele is present at the *REN11* locus (Zou et al., 2020), and an alternative hypothesis that each allelic site at the *REN11* locus contains the resistant allele. We converted the genotype to a 0/1 code, where 0 stands for the susceptible allele, and 1 stand for the resistant allele. Then, the hypothesis can be written as H0 ~ B (n0, p0) and H1~ B (n1, p1), where B (n, p) is a binomial distribution, n denotes the total number of allelic sites in this locus, p stands for the probability of the sites with desired alleles. The ratio of the likelihood of the alternative hypothesis to the null hypothesis is indicated by the Bayes Factor (BF). Above 1, larger BF values indicate greater support that the resistant alleles are present in this locus. The ranked log (BF) follows a sigmoidal distribution with *REN11*+ individuals having large values, *REN11–* individuals having small values and between them a steep slope in ranked log (BF) among full siblings with recombinations at the locus and related accessions lacking the complete *REN11* haplotype. The maximum differential of ranked log (BF) corresponded with BF values in the range of 4,094–10,266. This range included two individuals: *Vitis shuttleworthii* 2366 (BF = 5,685) and resistant progeny Y533_224 (BF = 7,727), so a BF threshold of 7,000 was set between them.

## Results

### Vineyard Phenotypes

**Figure 1:**
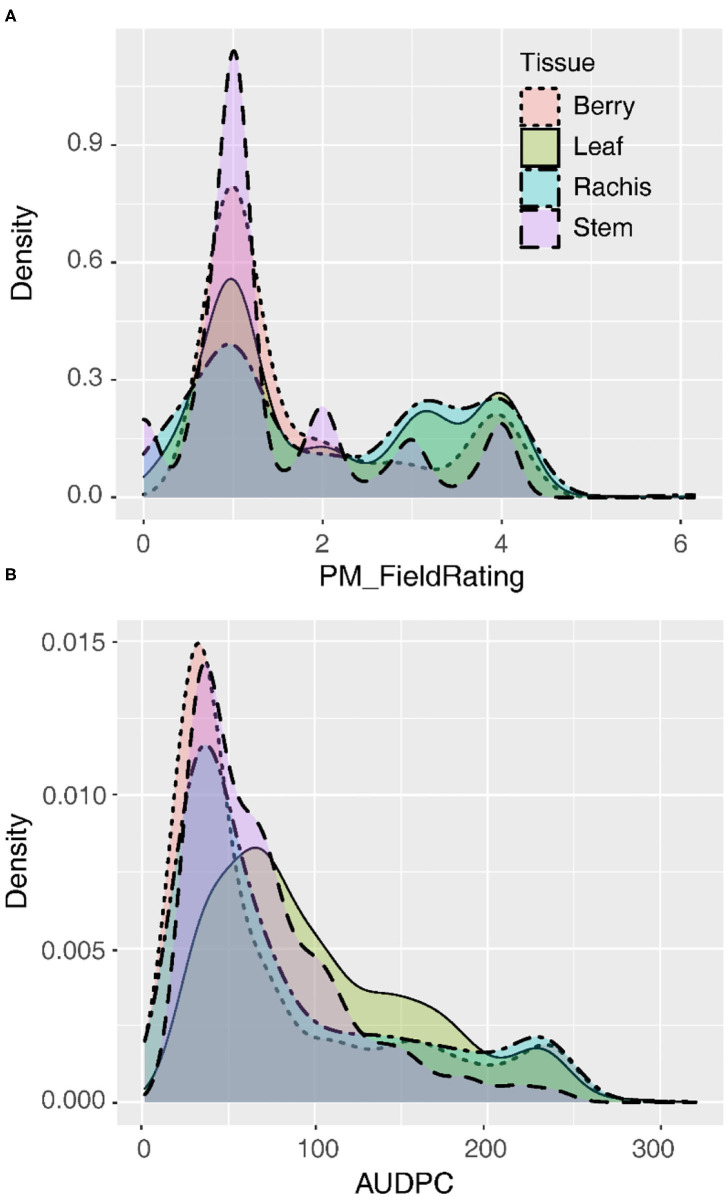
Distribution of disease severity values on four tissues summarized as the best linear unbiased estimates (BLUEs) for **(A)** qualitative field ratings (1–4) or **(B)** quantitative area under the disease progress curve (AUDPC).

Following natural infection in the vineyard, GPM severity rating distributions were skewed right regardless of whether the data were recorded on a 1–4 severity scale or AUDPC (Figure 1). The mode for the 1–4 severity scale was 1, indicating most commonly vines had no visible infection on all tissues observed. Similarly, for AUDPC, the mode for all tissues indicated vines most frequently had minimal GPM on any tissue, but leaves were slightly less skewed than other tissues. Susceptible vines had quantitative variability in disease severity on most tissues (Figure 1). Quantitative AUDPC was highly correlated among rachises and berries (*r* = 0.90–94) and well-correlated among leaves and those tissues (*r* = 0.61–0.84). In contrast, while stems had a similar intensity of disease severity as compared to the other tissues, stems were not as well-correlated with other tissues (*r* = 0.28–0.53).

### Genetic Map Summary

**Figure 2:**
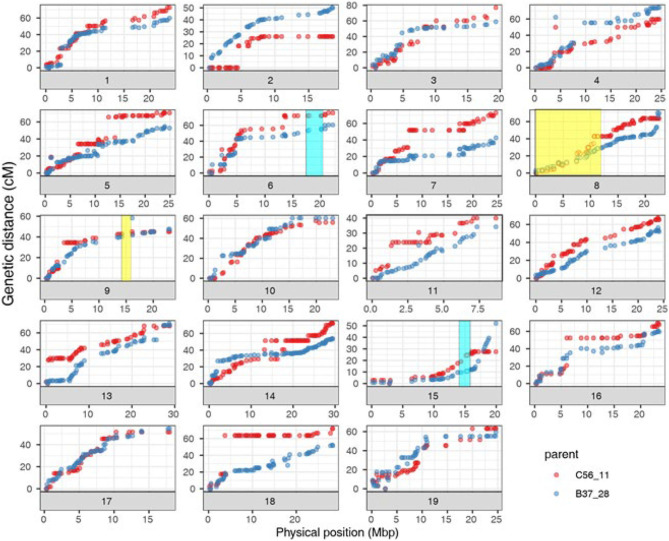
Correlation of B37-28 × C56-11 parental genetic maps and PN40024 12X.v2 physical maps for the 19 chromosomes (Chr). Four quantitative trait loci (QTLs) that were found two or more times are shown as transparent boxes in yellow for stems (Chr 8 and 9) and blue for all four tissues (Chr 15) or all tissues except berries (Chr 6).

The curated genetic map consisted of 1,058 rhAmpSeq core genome markers, forming 19 linkage groups after filtering for non-informative and distorted markers (Supplementary Tables 3, 4). The genetic map sizes of the parents ranged were 1,084.2 cM for B37-28 and 1,151.4 cM for C56-11. As with previous rhAmpSeq maps, both the maps were highly correlated to the physical map from PN40024 12X.v2 (Zou et al., 2020), with an average genome-correlation coefficient (*r*) of 0.92 and covering 93.4% of the genome physical distance (Figure 2, Supplementary Table 3).

### QTL Analysis

**Figure 3:**
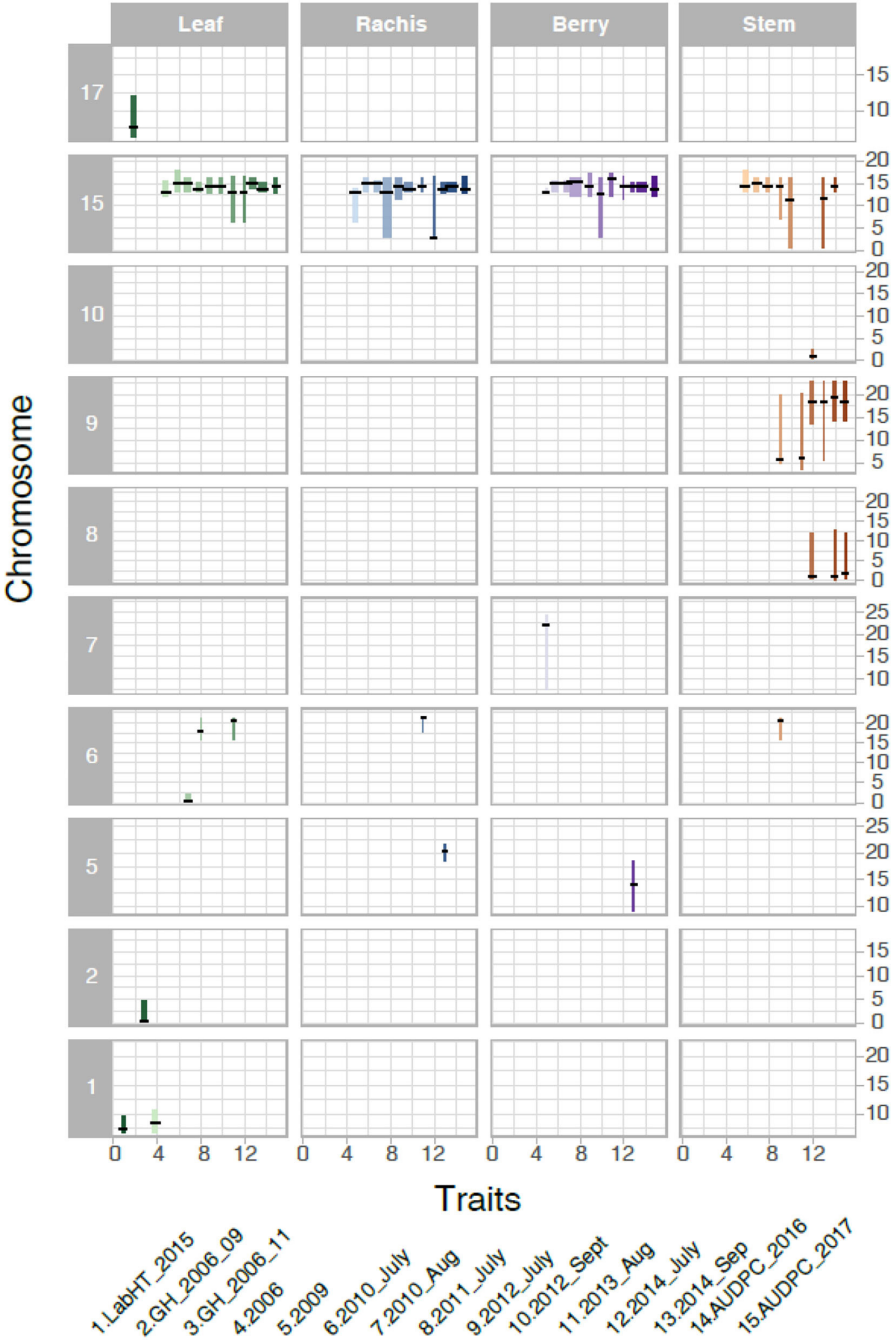
Significant Quantitative trait locus (QTL) for powdery mildew severity by tissue in B37-28 × C56-11. The 15 powdery mildew traits (or environments) analyzed correspond to numbers on the x-axis, with traits 4–13 corresponding to vineyard disease ratings on a 1–4 scale. The vertical interval of each line shows the chromosome Mbp region of the QTL, with a crosshatch at the QTL peak. The thickness of the line indicates the percent variance explained by the peak marker.

We analyzed the genetics of resistance in the vineyard, greenhouse, and laboratory. A locus from B37-28 on the chromosome (Chr) 15 near 13–15 Mbp was consistently detected on all vineyard-rated tissues in most years, explaining up to 96.2% of the phenotypic variation on the berries (Table 1, Figure 3, Supplementary Table 5). The locus was assigned the name *REN11*. Only in the first year of the study (2006, a year with data for only 49 vines, Supplementary Table 5) did *REN11* not significantly predict foliar vineyard severity; in 2006, Chr 1 near 7–10 Mbp was the only significant locus, explaining 38.0% of phenotypic variation on leaves.

**Table 1:** The number of vineyard environments, greenhouse (GH) timepoints, and laboratory (Lab) experiments with significant quantitative trait locus (QTL) for powdery mildew severity by tissue in B37-28 × C56-11.

		**Environments significant**					
**Chr^a^**	**Minimum Interval (Mbp)^a^**	**Leaf^b^**	**Rachis^b^**	**Berry^b^**	**Stem^b^**	**Maximum variance^c^**	**Resistance source**
**Validated^d^**							
6	17.68–21.33	2	1	–	1	5.2%	Either^e^
8	0.47–11.99	–	–	–	3	29.8%	B37-28
9	14.19–16.09	–	–	–	6	25.4%	B37-28
15	13.06–15.16	11	11	11	7	96.2%	B37-28
**Not Validated**							
1	6.87–10.67	1	–	–	–	38.0%	Either
1	6.87–9.70	Lab	–	–	–	27.9%	Either
2	0.39–4.60	GH	–	–	–	37.7%	C56-11
5	9.10–18.63	–	1	1	–	21.1%	Either
6	0.50–2.03	1	–	–	–	31.5%	Either
7	7.44–24.51	–	–	1	–	19.2%	Either
10	0.42–2.60	–	–	–	1	13.4%	Either
17	6.20–12.16	GH	–	–	–	39.1%	Either

*The bottom eight QTL were observed in only one environment and are considered not validated*.

a *Physical position in the PN40024 12X.v2 reference genome for the narrowest QTL identified for any significant environment; all significant QTL that overlaps this region are reported in this line. Chr, chromosome*.

b *Number of vineyard environments for which the QTL was significant for each tissue, with a maximum of 12 possible. Lab, controlled laboratory inoculation experiment; GH, natural inoculum greenhouse experiment with the early and late ratings*.

c *The maximum phenotypic variance explained by a QTL at this locus for a single environment*.

d *The top four QTL are considered validated because they were statistically significant in multiple independent environments*.

e *Either = Significantly increased susceptibility at this locus required a specific haplotype contributed from each parent. While Chr 1 QTL overlap, these susceptibility effects were in repulsion, meaning the opposite haplotype combination was required for increased susceptibility in the vineyard vs. the lab*.

**Figure 4:**
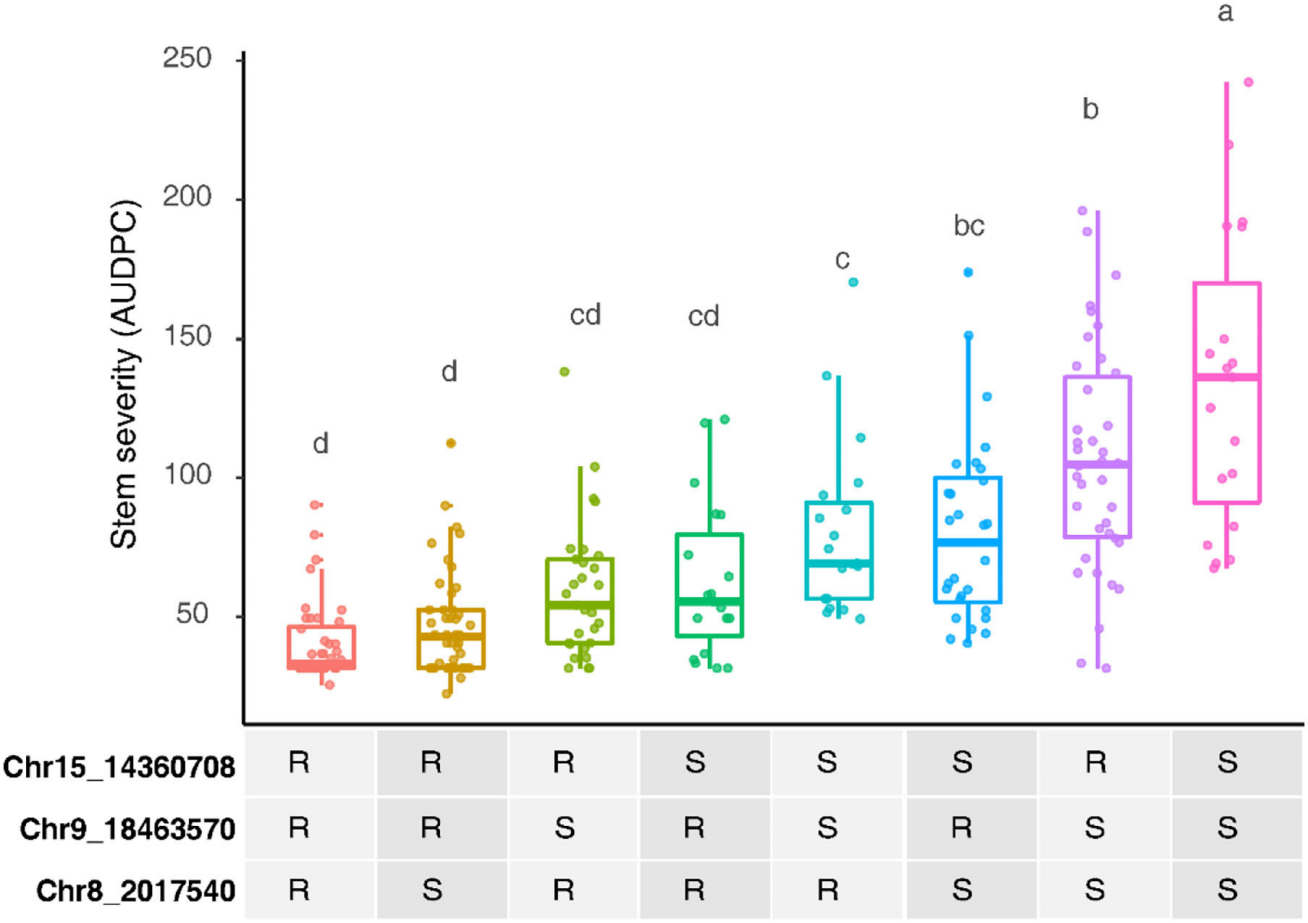
Additive effects of the powdery mildew resistance alleles on stems in B37-28 × C56-11, assessed by area under disease progress curve (AUDPC) BLUEs for 2016 and 2017 combined. For the three peak markers, R, resistant haplotype and S, susceptible haplotype. Letters above each box indicate statistically significant differences detected by Fisher's least significant difference test with Bonferroni corrections.

Three other loci were detected in multiple environments. On leaves, proximal Chr 6 (0.50–2.03 Mbp) explained 31.5% of the phenotypic variance late in 2010 and was epistatic with *REN11*, but distal Chr 6 (17.68–21.33 Mbp) explained less variance in 2011 and 2013 as well as on the single case when it significantly affected rachis or stem severity. In all the significant cases for Chr 6, a specific haplotype from each parent was required for significance, with that haplotype combination predicting increased susceptibility. Two loci from B37–28 repeatedly explained significant variation in stem severity: Chr 8 (3 ratings) and 9 (6 ratings). In quantitative AUDPC ratings, these two loci and *REN11* had additive effects in shifting mean stem GPM severity lower (Figure 4).

Six other QTL were detected in a single environment: two of these were from the Parlier greenhouse ratings that contributed to the hypothesis tested in this study (Ramming et al., 2012), each being significant at a different stage of the epidemic. Chr 2 was the only QTL with reduced severity inherited from C56-11, and explained differences late in the greenhouse epidemic, 2 months after the initial ratings. Epistatic interactions were only significantly detected in 1 year each for leaves, berries, and stems.

**Figure 5:**
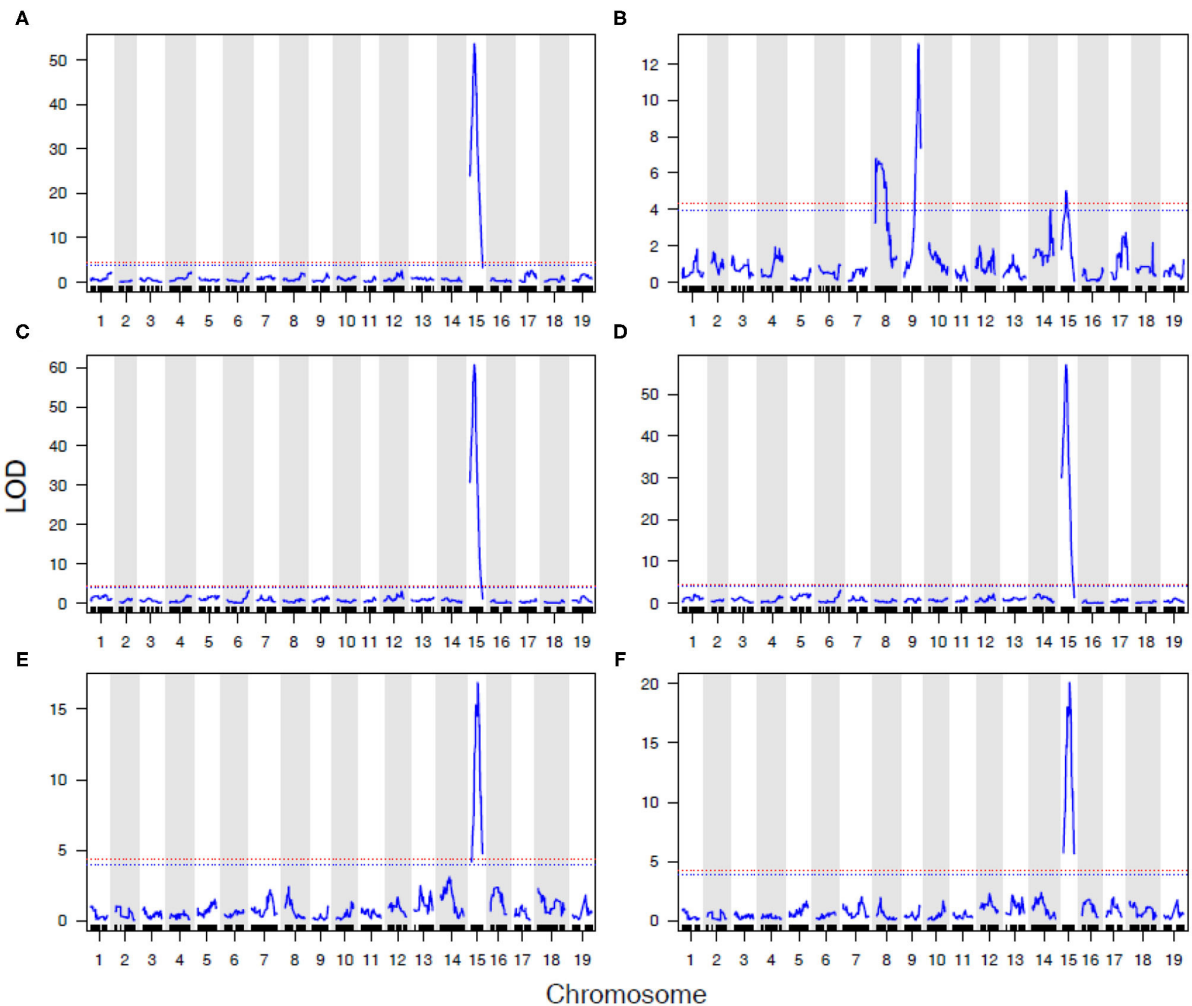
Powdery mildew resistance on four grapevine tissues repeatedly maps to *REN11* on Chr 15, except for a more complex genetic basis on B37-28 stems. Data for **(A–D)** B37-28 × C56-11 are based on AUDPC BLUEs for 2016 and 2017 combined, and for **(E,F)** Tamiami × Solbrio are based on 2017 AUDPC data. Tissues rated are: **(A,E)** leaf; **(B,F)** stem; **(C)** berry; **(D)** rachis. The 95% confidence threshold for the LOD score is based on permutation tests and is presented as a horizontal red dashed line.

In 2016 and 2017, a detailed AUDPC monitoring was conducted, and results reflected the observations in most years that *REN11* on Chr 15 significantly and strongly predicted disease severity on leaves, rachis, and berries, but additional loci were involved in resistance on stems (Figure 5). *REN11* was independently validated in the “Tamiami” × “Solbrio” AUDPC (Figure 5).

### Analysis of Recombinants at *REN11*

**Figure 6:**
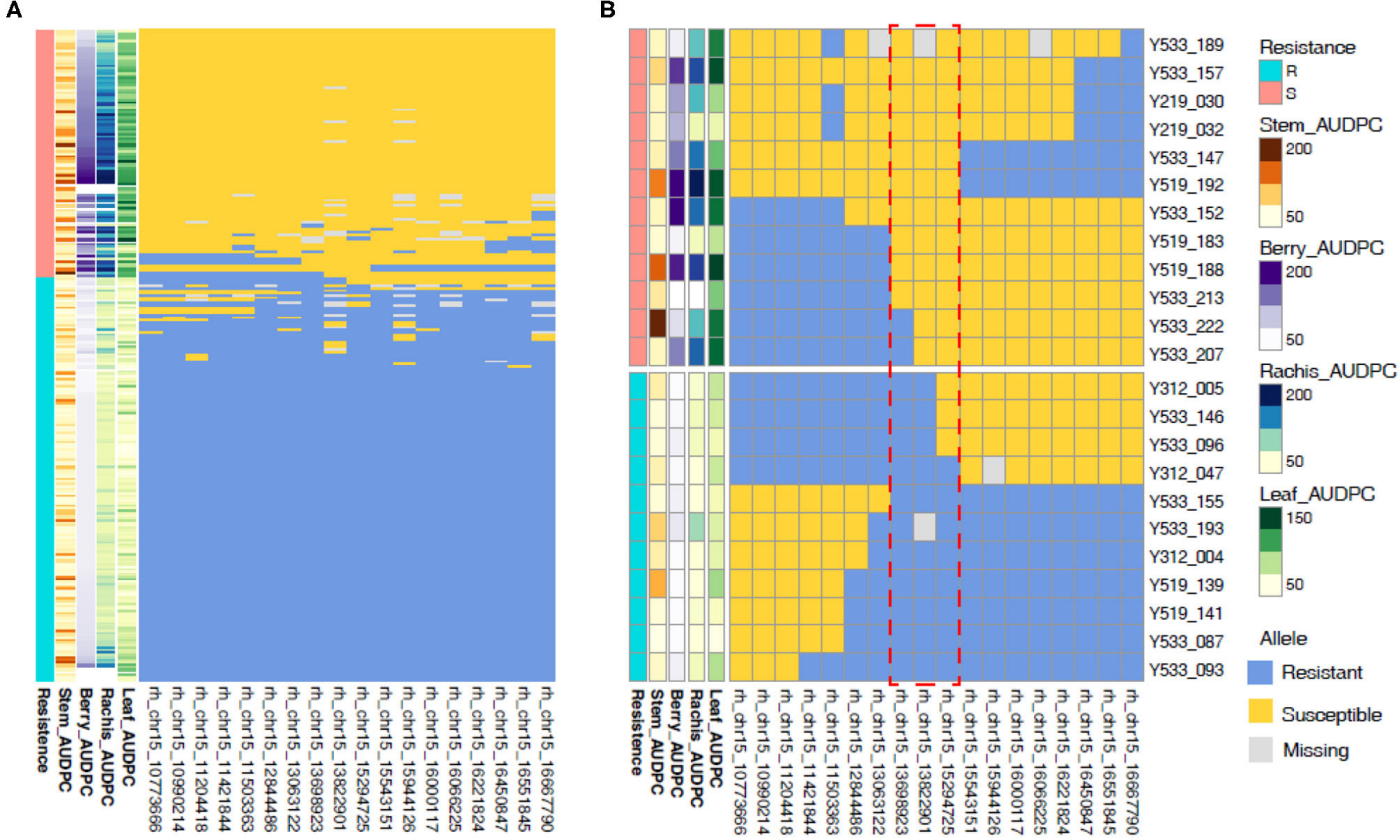
Recombinations within the *REN11* resistance QTL define the locus to 13.7–15.3 Mbp on Chr 15. Resistance was categorized subjectively based on the multi-tissue area under the disease progress curve (AUDPC) phenotypes from 2016 **(A)** across all samples, then **(B)** focusing on samples with recombinations in the *REN11* resistance QTL. Each row represents a different vine, and columns represent a phenotype or rhAmpSeq marker ordered by physical position. The red dotted box indicates the recombinations that define the locus.

To narrow in on the genetic basis of *REN11* resistance, we identified seedlings with recombinations within the QTL in relation to AUDPC phenotype from 2016. rhAmpSeq markers delimited the region to 13.7–15.3 Mbp on Chr 15, with the marker 15_13822901 being the most predictive of disease severity (Figure 6). This region of the reference PN40024 12X.v2 genome contains 149 genes including an NLR (nucleotide-binding, leucine-rich-repeat) resistance gene (VIT_215s0048g00680) that was previously shown to be upregulated upon inoculation of susceptible grapevines (Lin et al., 2019).

### Additional *REN11* Germplasm and Markers for Selection

The resistance alleles shown in Supplementary Table 6 can be used directly for the marker-assisted selection by AmpSeq. To identify additional germplasm with the *REN11* resistance haplotype, we screened the existing *Vitis*Gen database of 20,651 vines for these desirable resistance alleles. Based on a BF threshold of 7,000, 396 vines were identified as having the REN11 locus. These included the resistant progeny described in this study, 28 breeding lines (of which 25 are known to be related to Tamiami and two are from a breeder who frequently used Tamiami), and four interspecific hybrid accessions from the ARS repository at Winters, CA (Supplementary Table 7). These three breeding lines and four accessions represent a false positive rate of <0.034% (7 of 20,651). However, the four accessions differ in their rhAmpSeq haplotype at five markers (Chr15_12844486, Chr15_13063122, Chr15_15543151, Chr1515944126, and Chr15_16066225) flanking the *REN11* locus defined in Figure 6, suggesting that they do not have the *REN11* resistance allele. Thus, while the 18 haploblock markers presented in Figure 6 work well for the statistical prediction of *REN11* and identification of recombinant siblings, these five markers work well for eliminating rare false positives in a diversity panel.

### Analysis of Recombinants at Stem Resistance Loci on Chr 8 and 9

**Figure 7:**
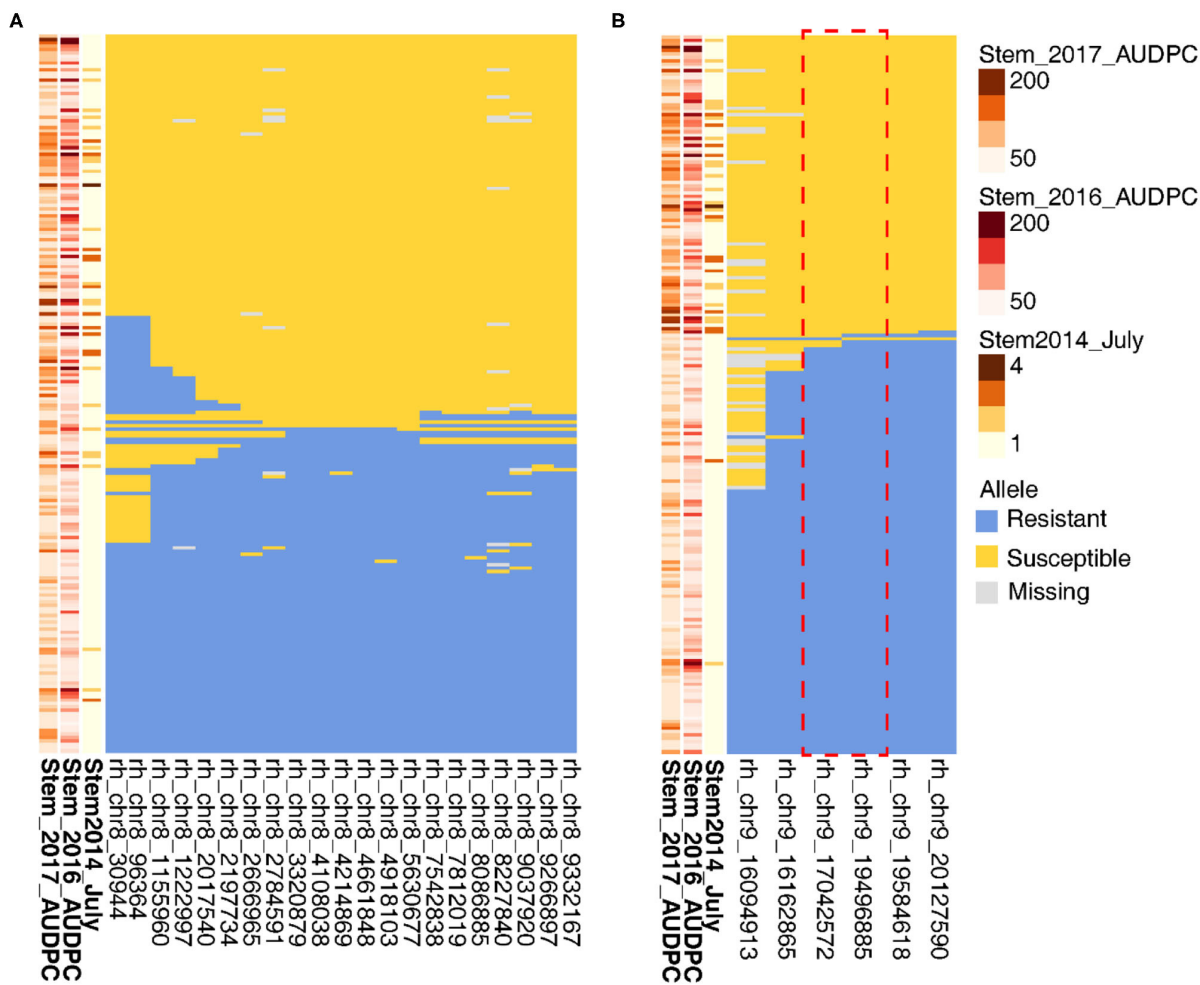
Recombinations within stem resistance QTL define the loci on Chr 9 but not on Chr 8. On the left of each panel, higher disease severity is indicated by the darker brown for **(A)** Chr 8 and **(B)** Chr 9. Each row represents a different vine, and columns represent a phenotype or rhAmpSeq marker.

To narrow in on the genetic basis of stem resistance, we identified seedlings with recombinations within the QTL. Because of the complex inheritance of the stem resistance, this approach failed to define the Chr 8 locus (Figure 7). For six key recombinant vines in relation to July 2014 phenotype, rhAmpSeq markers delimited the Chr 9 region to 17.0–19.5 Mbp (Figure 7), which is adjacent to an NLR locus in PN40024 12X.v2.

## Discussion

This study tested a previously published hypothesis that “Tamiami” confers three or more independently assorting, race-specific loci for GPM resistance (Ramming et al., 2012). Three foliar resistance QTL were discovered using data from Ramming et al. (2012), each explaining 37.7–39.1% of the phenotypic variance in those 2006 traits. However, none of those QTL was validated in the subsequent decade of the vineyard phenotyping. This could reflect the known race-specificity of resistance loci in this family, where controlled inoculation with *V. aestivalis*-derived isolates sporulated on otherwise resistant vines (Ramming et al., 2012).

Surprisingly, a different locus (named *REN11*) from “Tamiami” and “B37-28” (its resistant F_1_ offspring) conferred moderate-to-strong, qualitative GPM resistance in the vineyard every year after 2006. This locus spanning 13.7–15.3 Mbp on Chr 15 does not overlap with previously mapped GPM resistance loci on Chr 15 (REN3 near 9.3 Mbp and REN9 near 2.4 Mbp; Zendler et al., 2021), but its physical position may overlap with *RPV23* and *RPV26* from *Vitis amurensis*, conferring resistance to grapevine downy mildew caused by *Plasmopara viticola* (Lin et al., 2019; Fu et al., 2020).

*REN11* was uniformly effective and stable across years on leaf, rachis, and berry tissues, and it was validated in a second biparental family. In contrast, on stems, *REN11* explained phenotypic variance in 7 of 12 vineyard ratings, with QTL from “B37-28” on Chr 8 and Chr 9 frequently contributing to resistance with the additive effects. The average QTL for stems explained much less phenotypic variance (18.9%) than for leaves, rachis, and berries (33.4, 40.0, and 36.7%, respectively), reflecting the more complex genetic basis of resistance on stems in the B37-28 × C56-11 family. On Chr 9, we identified either genotyping error or structural variation associated with marker 9_16094913, which was genetically mapped three times as the upper bound of a QTL interval in which the peak marker was 9_18463570 or 9_19496885. While the authors have anecdotally observed that most GPM resistance loci seem to be similarly effective on all tissues, the ability of powdery mildew to colonize stems of otherwise resistant vines is reminiscent of powdery mildew colonization of *RUN1*+ stems (Cadle-Davidson et al., 2016).

The rhAmpSeq haplotype markers predicting *REN11* worked impressively well in the blind discovery of additional germplasm with *REN11* in a diversity panel of 20,651 vines representing the diversity of *Vitis*. This haplotype had a false positive rate of 0.034% or less, depending on the result interpretation. Of three *REN11*+ breeding lines with no known relationship to Tamiami, two came from a breeder (Zender) who commonly used Tamiami. Four hybrid accessions in the repository were statistically significant for *REN11*, but visual inspection of their haploblock alleles revealed that 5 of the 18 markers in the haploblock could specifically diagnose and distinguish the *REN11* haplotype studied here from rare false positives. At least 16 known wild *V. aestivalis* accessions and all the interspecific hybrids in the ARS-Geneva collection tested negative for *REN11* suggesting the resistant haplotype arose after species divergence. This is consistent with the variability in natural disease severity across wild *V. aestivalis* accessions in the vineyard (Cadle-Davidson et al., 2011a,b).

What about the race specificity of QR? That is difficult to address without additional controlled inoculations. In total, this phenotypic data set identified 11 significant QTL for GPM resistance in the biparental family B37-28 × C56-11. Aside from *REN11*, significant QTL explained between 2.9 and 39.1% of the phenotypic variance, and QTL detected more frequently did not explain more phenotypic variance. Chr 6, which was detected four times, explained only 2.9–5.2% of the phenotypic variance, so its infrequent detection may be because of the small effects that are difficult to detect. In contrast, two loci explaining more phenotypic variance Chr 9 (8.4–25.4%) and Chr 8 (9.0–29.8%) significantly predicted stem severity half the time or less despite being quantitative loci of moderate effect size. But targeted experiments are needed to determine whether those loci represent race-specific, QR loci. We had expected that a more quantitative assessment of disease severity represented by AUDPC would enable the identification of additional resistance QTL, but AUDPC results were strikingly similar to results from 1 to 4 ratings after 2006. We speculate that several years of pathogen adaptation to this resistance source may have enabled selection for virulence on the foliar resistance loci other than *REN11*.

In the GPM pathosystem, the null hypothesis is that dominantly inherited resistance phenotypes are conferred by the race-specific NLR genes. Indeed, most GPM resistance loci map to NLR regions including *REN11* and stem resistance on Chr 9, but not stem resistance on Chr 8. Still, NLR genes may be present on the Chr 8 locus in the *V. aestivalis* genome, which warrants further investigation.

The project presented in this study spanned 15 years, creating considerable challenges for summarizing the results. Available B37-28 × C56-11 samples changed over time resulting in imperfect overlap of the phenotypic and genotypic results due to moving the vines to a new site, vine death, and new seedlings being grown and phenotyped at two locations, Parlier, CA, and Geneva, NY. Furthermore, marker technologies and genome assemblies evolved considerably, resulting in the following unpublished data. We first detected the effects of Chr 15 *REN11* using SSR markers in 2009, an effort that took 1 year to obtain an imperfect linkage map of just over 100 markers. We then used the Vitis9KSNP array (Myles et al., 2010), which at $150 per sample and limited availability could only be applied to a small subset of seedlings; still, we detected the effects of Chr 6 (18.5-19.8 Mbp on PN40024 8x assembly) and Chr 15 (5.4–7.1 Mbp on PN40024 8x assembly) for phenotypes from natural infection in a Geneva, NY greenhouse, not presented in this study because DNA was not archived and available for rhAmpSeq. Genotyping-by-sequencing (GBS) confirmed Chr 15 (13.4–14.8 Mbp in PN400024 version 12X.0) for the Geneva, NY, greenhouse phenotypes, but only a subset of the vines analyzed in this study were genotyped with GBS. Because SNP markers (e.g., GBS and Vitis9KSNP) are not transferable in *Vitis*, and because of the complexity of matching the aforementioned older genotypes and physical coordinates from previous reference genomes with phenotypes, we decided to simplify in this study and present only the data that could be analyzed with the rhAmpSeq local haplotype markers that US grape breeders are currently using for marker-assisted selection and linkage mapping. Even with that simplification, organizing, analyzing, and summarizing 53 GPM phenotypes (12 environments × four tissues, two greenhouse timepoints, a lab experiment, and two AUDPC tissues from a second family) collected over 12 years by three breeders, five postdocs, and several technicians (most of whom no longer conduct grape research) was a large and complicated task. However, the results with the previous marker platforms particularly phenotype not analyzed with rhAmpSeq, further substantiate the stability and strength of *REN11* across diverse environments, and the moderate contribution of distal Chr 6 across diverse environments.

In summary, in this study, we describe a novel locus *REN11* conferring reproducibly strong powdery mildew resistance from “Tamiami,” and its resistant F_1_ progeny “B37-28,” which should be a valuable resource for powdery mildew resistance breeding. Resistance on stems is more complex than on leaf, rachis, and berry tissues, with *REN11* and loci on Chr 8 and 9 conferring QR in some of the environments. Sequences spanning these three haploblocks are provided for selection in rhAmpSeq or conversion to other marker platforms. Chr 6 has another promising locus for foliar GPM resistance, but other foliar resistance loci detected in this study too seem to be unstable for genetic improvement regardless of quantitative effect size, whether due to race specificity or other environmental variables.

## Data Availability Statement

The datasets presented in this study can be found in online repositories. The names of the repository/repositories and accession number(s) can be found at: https://www.ncbi.nlm.nih.gov/, bioproject PRJNA281110.

## Author Contributions

DR, RN, and CL developed the germplasm. AK and CZ performed the analyses presented in this study. LC-D wrote the first draft of the manuscript, with methods sections contributed by AK, CZ, and RN. All the authors contributed to data collection and preliminary analysis, manuscript revision, and approved the submitted version.

## Conflict of Interest

The authors declare that the research was conducted in the absence of any commercial or financial relationships that could be construed as a potential conflict of interest.

## Publisher's Note

All claims expressed in this article are solely those of the authors and do not necessarily represent those of their affiliated organizations, or those of the publisher, the editors and the reviewers. Any product that may be evaluated in this article, or claim that may be made by its manufacturer, is not guaranteed or endorsed by the publisher.
